# Exploring Practice Approaches and Opinions of International Speech and Language Therapists in the Assessment and Management of Oropharyngeal Swallowing in Adults on High‐flow Nasal Oxygen: A Focus Group Study

**DOI:** 10.1111/1460-6984.70333

**Published:** 2026-09-08

**Authors:** Andrea Hanratty, Claire Mills

**Affiliations:** ^1^ Department of Clinical Speech and Language Studies Trinity College Dublin Dublin Ireland; ^2^ Speech‐Language Therapy Department Auckland City Hospital Auckland New Zealand; ^3^ Speech and Language Therapy Heart Lung and Critical Care Royal Brompton and Harefield Hospitals Guy's and St. Thomas’ NHS Foundation Trust London UK; ^4^ Academic Unit of Health Economics Leeds Institute of Health Science University of Leeds Leeds UK

**Keywords:** assessment, critical care, high flow nasal cannula, high flow nasal oxygen, oropharyngeal swallowing, qualitative

## Abstract

**Background:**

Speech language therapists (SLTs) are recognised as key members of the multidisciplinary team in adult critical care and have more recently been assessing and managing swallowing with adults on high‐flow nasal oxygen (HFNO). HFNO is a non‐invasive respiratory support that delivers a stable fraction of inspired oxygen ranging from 0.21 to 1.0 with flow rates of 15–60 litres per min. Despite HFNO being used routinely in critical care and SLTs postulating an effect of HFNO on oropharyngeal swallowing, there is limited evidence and consensus of potential effects in the literature. Subsequently, there are no clear guidelines or practice patterns for managing dysphagia in this patient cohort. There is limited understanding of how SLTs are interpreting and applying the available literature to their practice patterns.

**Aim:**

The aim of this qualitative study was to explore international SLTs’ practice approaches and opinions when assessing and managing oropharyngeal swallowing in adults on HFNO.

**Methods & Procedures:**

Participants were SLTs with at least two years of experience working in critical care. Three online focus groups consisting of 18 participants from six countries were conducted. The participants were asked about their current practices, opinions, considerations, and perceptions of challenges and opportunities when assessing and managing adults on HFNO. Inductive content analysis was used to analyse the data.

**Outcomes & Results:**

Six content categories were identified; ‘consider the patient individually and holistically’, ‘types of assessment procedures’, ‘impact of HFNO on oropharyngeal swallowing’, ‘considerations when assessing adults on HFNO’, ‘current knowledge and practice landscape’, and ‘collaboration and communication’. A further 15 subcategories were derived.

**Conclusion:**

This research has identified concerns amongst SLTs that there is a lack of evidence to guide assessment and management of dysphagia in patients receiving HFNO. Research efforts should focus on clarifying how different flow rates impact swallowing function. This would help establish best‐practice approaches for screening, assessing, and managing swallowing in patients receiving HFNO.

**WHAT THIS PAPER ADDS:**

*What is already known on this subject*

- High‐flow nasal oxygen (HFNO) is being used more frequently to treat critically‐ill adults, however the impact of HFNO on oropharyngeal swallowing has not been fully established. Speech and language therapists (SLTs) are now routinely integrated into the multidisciplinary team in critical care and assess adults on HFNO. To the authors knowledge, no study has previously explored in‐depth the perspectives of critical care SLTs who assess and manage oropharyngeal swallowing in adults on HFNO.

*What this paper adds to the existing knowledge*

- This is the first qualitative study exploring international SLTs opinions and practices when assessing and managing oropharyngeal swallowing in adults on HFNO. This study provides valuable insights into how oropharyngeal swallowing is currently being assessed and managed internationally by SLTs and provides guidance on future research in this area.

*What are the potential or actual clinical implications of this work?*

- Findings from this study demonstrate variability in clinical practice and highlights uncertainty regarding assessment and management in this patient population. Some participants emphasised the value of instrumental assessment in supporting clinical decision making. This study identified that there are currently no standardised protocols or guidelines for the screening and comprehensive assessment for adults on HFNO. Further research is needed to guide the development of such protocols and guidelines to enable best practice.

## 1 Introduction

Speech and Language Therapists (SLTs) treat people with swallowing and communication disorders and are recognised as key members of the multidisciplinary team (MDT) in the critical care setting (McRae et al. 2020). HFNO is a type of non‐invasive respiratory support, which in recent years has been used to treat critically‐ill adults in respiratory distress (Nishimura 2015). HFNO delivers a stable fraction of up to 1.0 of inspired oxygen (FiO_2_) at flow rates of 15–60 litres per min (L/min) (Nishimura 2015, Fisher and Paykel 2025, Nishimura 2016, Rochwerg et al. 2019). A study found that healthy adults have a mean peak inspiratory flow rate (PIFR) of 27.9 L/min at rest, while adults with pulmonary disease have a higher PIFR of 34 ± 9 (Ritchie 2011, Li et al. 2021, Li et al. 2023). The therapeutic benefits of HFNO occur when the gas meets or exceeds the patient's inspiratory flow requirement (Li et al. 2020, Li et al. 2023). The amount of FiO_2_ and flow rate used for HFNO delivery is determined by the degree of hypoxemia, inspiratory effort, and patient comfort (D'Cruz et al. 2020, Li et al. 2023).

Specific patient populations where HFNO may be indicated include pre‐ and post‐extubation, community‐acquired pneumonia, acute pulmonary oedema, acute hypoxemic respiratory failure (AHRF), chronic obstructive pulmonary disease or where non‐invasive positive pressure ventilation is not tolerated (Rochwerg et al. 2020, Feng et al. 2022, Lodeserto et al. 2018). There are many beneficial mechanisms of action or physiological effects of HFNO including: increased positive end expiratory pressure (PEEP), carbon dioxide washout in the anatomical dead space, improved mucociliary clearance, increased patient comfort through humidification, alveolar recruitment, and reduction of nasopharyngeal resistance (Nishimura 2015, Lodeserto et al. 2018, Gotera et al. 2013).

A systematic review and meta‐analysis including randomized controlled trials investigating the use of HFNO in patients with AHRF found that use of HFNO may decrease the necessity for tracheal intubation compared with conventional oxygen therapy (Rochwerg et al. 2019). A more recent systematic review by Li et al. (2023) investigated the effects of flow rates in adults receiving HFNO therapy and found that physiological effects are dependent on the flow rate. It was concluded that higher flow rates can reduce work of breathing, improve dead space washout, and improve oxygenation. The authors also highlighted the importance of individualising flow rates and considering patient comfort alongside adjusting flow based on oxygenation and respiratory rate. One of the benefits of HFNO is that the person's mouth is unrestricted which can allow eating, drinking, and speaking compared to traditional oxygen therapy and continuous positive airway pressure (CPAP) where a mask or hood covers the oral cavity (Ward 2013).

Respiration and swallowing are intrinsically linked by their shared anatomical pathway (the pharynx). In healthy adults there is a stable coordination of these functions (Martin 1994, Martin‐Harris 2005, Martin‐Harris 2008, Matsuo and Palmer 2009). For example, when healthy individuals are swallowing liquids, there is a respiratory pause, also known as ‘swallow apnoea’, of approximately 1–1.5 s (Martin‐Harris 2005, Preiksaitis 1992). Evidence suggests that the typical respiration‐swallowing pattern in adults is ‘exhale‐swallow‐exhale’ or ‘inhale‐swallow‐exhale’. The exhalation post‐swallow is an airway protective mechanism to ensure that any material in the pharynx is expelled after swallowing (Matsuo and Palmer 2009). Despite this close relationship between respiration and swallowing, the physiological effects of HFNO on oropharyngeal swallowing have not been fully established (Coghlan and Skoretz 2017, Devlin and O'Bryan 2021).

The studies investigating oropharyngeal swallowing with HFNO have varied in population, method, data collection, and results. Although the literature on HFNO and oropharyngeal swallowing is inconclusive, the studies which use Videofluoroscopic Swallow Studies (VFSS) to measure physiological components of healthy individuals swallowing under different flow rates found that swallowing is altered (Allen and Galek 2020, Eng et al. 2019, Khan et al. 2022). Allen and Galek (2020) found that healthy individuals had an increased duration of laryngeal vestibular closure as flow rates increased, with greater variability at flow rates 50–60 L/min whereas Eng al. (2019) found that flow rates of 60 L/min impacted the oral phase of swallowing. Khan et al. (2022) (abstract only) used Flexible Endoscopic Evaluation of Swallowing (FEES) to assess eight healthy individuals swallowing with different flow rates (0, 30, 45 and 60 L/min) and found that with higher flow rates there was a significant increase in premature pharyngeal entry, increased vallecula and pyriform sinus stasis, and an increase in penetration events. Conversely, Graf et al. (2024) assessed 26 healthy individuals using FEES with flow rates of 0, 30, 40, 50 and 60 L/min and found increasing flow rates do not impact on swallowing safety. However, studies of healthy participants’ oropharyngeal swallowing may not be reflective of the patient populations in a hospital setting.

There have been few studies which investigate adult patients on HFNO using instrumental assessment. A recent retrospective study of 21 patients investigated swallowing under different flow rates (15–30 L/min) using FEES, finding that swallowing function was significantly more impaired with high flow nasal cannula than without (Ellsworth and Bartlett 2025). Another retrospective study reviewed 10 patients and assessed swallowing function on flow rates ranging from 30–50 L/min reporting silent aspiration in 50% of these patients. Despite this, all patients were commenced on oral intake, with the majority requiring texture modification and compensatory strategies (Flores et al. 2019). Leder et al. (2016) completed a prospective cohort study which included 50 neonates and 50 adults. Of the adult cohort, patients were receiving oxygen on flow rates ranging between 10–50 L/min. Adult patients were commenced on oral intake if they had a stable respiratory status, adequate cognitive status, and if they passed the Yale Swallow Protocol which includes a 3‐ounce water swallow test. Five patients failed the Yale Swallow Protocol and underwent a FEES which identified pharyngeal dysphagia, however, these patients were able to commence oral intake with diet and fluid modifications (Leder 2014). It is worth noting that the Yale Swallow Protocol has not been validated using instrumental assessment in patients receiving HFNO.

While there have been survey studies carried out on international practices for feeding while patients are receiving HFNO (Canning et al. 2020, Charlton et al. 2023), the opinions, experiences, and perspectives of SLTs on those practices have not yet been examined. Findings from this study will help prioritise research efforts toward what will be of most benefit to practising clinicians. This research aimed to explore international SLTs’ practice approaches and opinions when assessing and managing oropharyngeal swallowing in adults on HFNO. The objectives were to understand experiences and opinions; gain insight into current practice; explore considerations during assessment and management; and identify challenges and opportunities.

## 2 Methods

### 2.1 Researcher and Reflexivity

The Principal Investigator (PI) primarily works with adults with dysphagia in critical care. Prior to commencing the study, the PI's perception was that there were a high number of referrals to assess oropharyngeal swallowing for adults in critical care receiving HFNO. To date, there has been minimal research regarding the effect of HFNO on adult oropharyngeal swallowing, and to the authors’ knowledge, no qualitative research on how SLTs are assessing and managing adult patients on HFNO. These research gaps were the PI's primary motivation for undertaking this research project.

### 2.2 Study Design

A qualitative focus group methodology was chosen for this study. The consolidated criteria for reporting qualitative studies (COREQ) were used for the reporting of this research (Tong 2007). All participants provided informed consent prior to participating.

### 2.3 Participants and Sampling

**FIGURE 1:**
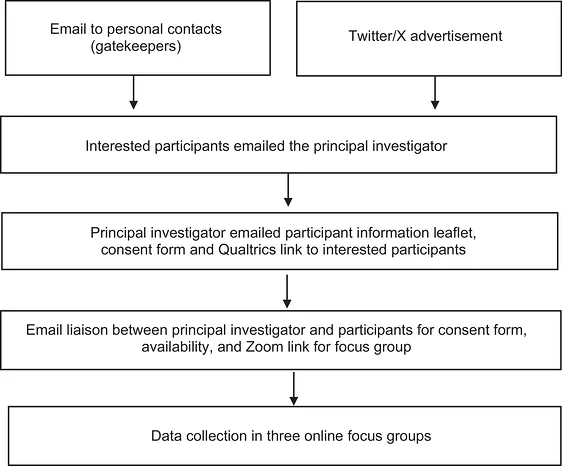
Recruitment process.

The target population were qualified SLTs with at least two years of clinical experience working in critical care, proficiency in spoken English, and ability to participate in an online focus group between 27^th^ March 2023 and 14^th^ April 2023. Recruitment through convenience and snowball sampling is described in Figure 1. Firstly, participants were selected based on the country of abode, as this study aimed to explore perspectives of SLTs working internationally. Participants were then selected based on their availability to attend the focus group sessions within the specified time frame. All participants received a participant information leaflet and provided written consent. The study aimed to recruit a minimum of 15 participants across three focus groups.

### 2.4 Data Collection

The focus group interviews were guided by pre‐determined questions which were simple, unbiased and open‐ended (Wong 2008). The semi‐structured interview guide was developed by the PI and feedback was received from the research supervisors. The discussion guide was emailed to the participants prior to the focus groups, and contained the following questions:

- What are your opinions of assessing and managing oropharyngeal swallowing in adults on HFNO?
- What considerations do you have when assessing and managing adults on HFNO?
- How is oropharyngeal swallowing in adults on HFNO assessed and managed in your setting?
- What do you perceive to be the challenges when assessing and managing oropharyngeal swallowing in adults on HFNO?
- What do you perceive to be the opportunities when assessing and managing oropharyngeal swallowing in adults on HFNO?

The focus groups took place on the ‘Zoom’ platform on the 26^th^ March 2023, 30^th^ March 2023, and the 12^th^ April 2023. Participants attended one focus group only. Participants were required to have their videos on for the duration of the focus group to allow for an interactive discussion. Audio recordings were saved to a password protected drive and data were transcribed verbatim by the PI. Data were pseudo‐anonymised, and audio recordings were deleted in line with European Union General Data Protection Regulation. Participants were advised they could request a copy of the transcript, however, no participants requested this.

Alongside the PI who facilitated the focus group, there were two note takers (FB and DB) in the first and second focus group, and one note taker (FB) in the third focus group. Note takers were present to ensure the participants were correctly identified for transcription.

### 2.5 Data Analysis

Given the limited existing knowledge in this area of research, it was determined that inductive content analysis was an appropriate analytic method as it supports the exploration of human perspectives and experiences (Elo and Kyngas 2007). Additionally, this method of data analysis is appropriate when the research is seeking practical information (Vears and Gillam 2022). The analytic process followed established stages of inductive content analysis: 1) read and familiarise; 2) first‐round coding: identifying big picture meaning units; 3) second‐round coding: developing subcategories and fine‐grained codes; 4) refining the fine‐grained subcategories; and 5) synthesis and interpretation (Elo and Kyngas 2007, Vears and Gillam 2022). Transcripts were coded manually rather than using qualitative analysis software. Manually coded transcripts were considered to be the more suitable method of analysis due to the small size of the dataset. This also allowed close engagement with the data. To ensure rigor and consistency, an audit trail was maintained throughout the analytic process. This involved annotated transcripts, evolving coding frameworks, and records of analytic decisions which enabled transparency in how findings were derived from the data. To confirm the synthesis of the content and subcategories, preliminary analyses and results were discussed with the research supervisors.

### 2.6 Participants

Eighteen participants from six countries contributed across the three focus groups (n = 6; n = 8; n = 4). Focus group duration ranged from 42– 53 min (Table 1). Focus group participant numbers were unevenly distributed due to availability and time zone differences. The participants’ years of experience working in critical care is described in Table 2. No other demographic information was collected or analysed.

**TABLE 1:** Focus group information.

Date of focus group	Focus Group Number	Country representation	Number of participants	Duration of focus group (minutes)
26/03/2023	FG1	Australia New Zealand	1 5	42
30/03/2023	FG2	United Kingdom Republic of Ireland United States of America	2 2 4	53
12/04/2023	FG3	United Kingdom Italy	3 1	42

Abbreviation: FG, Focus Group.

**TABLE 2:** Participant experience working in critical care.

Number of years	Number of participants
2–5 years	5
6–10 years	5
11–19 years	6
20+ years	2

## 3 Results

Following data analysis, six content categories and 15 subcategories were identified (Table 3). The categories and subcategories identified though inductive content analysis will be illustrated using verbatim quotes from the focus groups.

**TABLE 3:** List of content categories and subcategories.

Consider the patient individually and holistically Patient status and trajectory Readiness and timing of assessment Types of assessment procedures Clinical bedside swallowing assessment Instrumental assessment of swallowing Impact of HFNO on oropharyngeal swallowing Negative impact Potential to commence oral intake Considerations when assessing adults on HFNO Flow rate Respiratory‐swallow coordination Safety and efficiency Pressure changes Current knowledge and practice landscape Frequency of HFNO use Staffing challenges Lack of evidence and research Collaboration and communication Working with the multidisciplinary team Patient experience

### 3.1 **Category 1**:Consider the Patient Individually and Holistically

Participants universally expressed that when assessing and managing adults on HFNO, the patient should be considered individually and holistically. This revealed two subcategories: patient status and trajectory, and readiness and timing of assessment.

#### 3.1.1 Patient Status and Trajectory

The participants discussed the importance of patient status, highlighting that in critical care, patients are particularly vulnerable, medically fragile, and commonly have multiple comorbidities. The following quote is highly representative of the sentiments shared in each of the focus groups: “*If anyone is going to get sick from aspiration, it's probably our people who transition from high‐flow. They are still quite vulnerable and still largely bed bound” (FG1)*. Participants reported that management of patients are influenced depending on the patients’ location (critical care, ward) and their trajectory (improving or deteriorating).

#### 3.1.2 Readiness and Timing of Assessment

Participants reported considering where the patients were in their respiratory weaning journey when determining readiness for swallowing assessment. They reflected on when the most appropriate time for an assessment is, given that these critically‐ill patients’ conditions may fluctuate. The following quote was chosen as it reflects a common consideration by SLTs regarding timing of assessment: *“What is the purpose of the assessment if that patient is still actually not ready to eat and drink and doesn't want to eat and drink because they're so fatigued from just the respiratory effort?” (FG2)*.

Participants reported sometimes there is pressure from the MDT to assess patients soon after extubation. This quote was chosen as it captures the clinical dilemma around readiness and timing of swallowing assessments in this patient cohort: *“An hour post extubation we've got nurses chasing us wanting assessment but again we don't know which way they (the patients) will go—these patients are really on the knife edge at times” (FG1)*.

The following quote is a description of an interaction that one participant had when a member of the medical team suggested reducing the flow rate at the time of the swallowing assessment: *“Well fine … what is the utility of my swallow assessment?’ … you know that's slightly a pointless exercise if they're just going to whack that high flow back up when I've left the room” (FG2)*.

### 3.2 **Category 2**:Types of Assessment Procedures

None of the participants reported using a HFNO dysphagia screening tool, or any other formal dysphagia screening protocol. One participant identified the lack of consensus by SLTs about the appropriateness of screening patients on HFNO, this quote was selected because it captures an important aspect of this theme: *“Should we be screening? Shouldn't we? […] I don't know who's doing what in practice” (FG3)*. All participants reported using clinical bedside swallowing assessments, and there was variable use of instrumental assessment of swallowing.

#### 3.2.1 Clinical Bedside Swallowing Assessment

All participants reported that they assess adults on HFNO with a clinical bedside swallowing assessment. This included taking a thorough case history, completing a cranial nerve examination, and food and fluid trials. However, concerns were raised by participants about the reliability of bedside swallowing assessments in this population. The following quote clearly illustrates the concerns regarding this type of assessment: *“Bedside assessments can be really muddy and murky” (FG1)*.

#### 3.2.2 Instrumental Assessment of Swallowing

All participants reported that they work in hospitals which have access to instrumental assessment including FEES and VFSS. However, the usage and timing of these assessments varied widely, as demonstrated by the quotes chosen. Participants in Australia and New Zealand reported they often prefer to assess patients on HFNO using instrumental assessment: “*We would most likely go to an instrumental assessment […] we are quite a lot more confident using instrumental” (FG1*);*“We'd probably have a lower threshold for these people who were probably more vulnerable to go to FEES” (FG1)*. In other countries there were differing reasons given for not routinely using FEES with patients on HFNO. A participant from the United Kingdom reported that changes in medical status would preclude them from completing these assessments: *“Why we're not doing as much FEES? Because the patients fluctuate” (FG3)*. In Italy, having to rely on a different profession was reported to be a barrier: *“In critical care we need to ask for FEES and usually is [completed by] department of ENT [Ear Nose and Throat] […] it will take us a week to have the proper (FEES) examination” (FG3)*.

Participants who regularly use FEES to assess and manage adults on HFNO reported that it was common to observe residue in the larynx, and voiced concerns that HFNO was a contributing factor. One participant reflected on clinicians who are not routinely using instrumental assessment: *“Different practices and people who don't have access to FEES or video don't necessarily know what they don't know” (FG1)*, another stated: *“they don't have that image of that residue waving around in the larynx […] once you've seen it you can't unsee it” (FG1)*. These two quotes were chosen as they provided critical insights to this theme.

Benefits and barriers to using FEES or VFSS were discussed in all focus groups. Participants discussed that when using FEES there is missing visualisation of the oral phase and what happens during the swallow which is captured using VFSS. However, with VFSS multiple barriers were identified including the need for clinical stability, transport to Radiology, and staffing. A participant from New Zealand reported *“we do video(fluoroscopy) for people with high‐flow but it's a lot more difficult logistically just getting them down there [to Radiology] with oxygen” (FG1)*. Similar concerns were shared from a participant from the Republic of Ireland who said *“I would be more inclined to do videofluoroscopy as well probably if they're safe enough to leave the unit […] but very often those patients aren't clinically suitable to transfer to interventional radiology” (FG2)*.

### 3.3 **Category 3**:Impact of HFNO on Oropharyngeal Swallowing

Participants discussed the potential impact of HFNO on oropharyngeal swallowing, including both the negative impact and potential benefits. The quotes in this category were selected to represent the broad range of opinions across all three focus groups.

#### 3.3.1 Negative Impact

Participants hypothesised how HFNO may be interfering with various aspects of swallowing physiology with higher flow rates. One participant stated *“I think being super aware that things like the pressures, the sensation, residue, apnoeic stage might all just be impacted by having high flow” (FG1)*. Another participant said *“The higher the flow rate, the more I would be thinking about how it may impact their swallow. (FG1)”*

#### 3.3.2 Potential to Commence Oral Intake

Whilst there were hypothesised negative impacts in the above subcategory, some participants described potential positive impacts of HFNO on swallowing function. Enhanced respiratory support and humidification provided by HFNO were seen as possible contributors to improved swallowing function and comfort as demonstrated in this statement: *“Whether the flow does improve… in terms of whether it does help swallow function sometimes? Maybe? Yes, jury is out, I think” (FG3)*.

HFNO was also reported to enable earlier swallowing assessment, rehabilitation, and commencement of oral intake. Participants outlined the potential benefit of patients spending shorter periods nil by mouth. This was highlighted for both swallowing progression: *“The earlier we treat the patients the better—so we can speed up the recovery process” (FG 2)*, and patient comfort: *“I always […] go back to the […] quality‐of‐life element of it as well and the making sure that patients are comfortable and often, I find that particularly with high‐flow they want something—they want water or even if it's like tiny amounts. They want something to keep that swallow going” (FG2)*.

### 3.4 **Category 4**:Considerations When Assessing Adults on HFNO

Participants identified a wide range of clinical considerations when assessing and managing adults on HFNO. The following subcategories represent the aspects that were most frequently raised and discussed across all focus groups: flow rate, respiratory‐swallow coordination, safety and efficiency, and pressure changes.

#### 3.4.1 Flow Rate

Higher rates of flow were widely considered to have a negative impact on oropharyngeal swallowing. It was also discussed that clinical rationale varies across patient cohorts. The following two quotes demonstrate this:

“I think the higher […] flow they're on, the more I get concerned about their ability to compensate […] for my cohort in ICU [Intensive Care Unit] they are weak, there could be some difficulty in adapting their swallow to compensate for a high‐flow respiratory sort of air flow […]if they are too weak they can't necessarily adapt” (FG2).

“I probably won't go near anything above 30 litres but in my head that's not an example to be quoted in any kind of teaching. I'm very careful about not using that as a prescriptive thing but in my head, I just think, oh I know my client group here and I know that anything above 30 means that this patient group will need all their reserve for breathing” (FG2).

Another quote further highlights the difficulties and reliance on clinical reasoning to justify assessment on higher flow rates in the absence of robust evidence.

“You just have […] this 40 litres drilled into us and then when you try and go above and beyond it when especially when you got maybe more junior staff who are learning … numbers don't mean anything you have got to look at this is a whole and that is really hard if you're not as experienced clinically I think to kind of not justify why you're doing it but explain why you doing it” (FG3).

#### 3.4.2 Respiratory‐Swallow Coordination

When assessing adults on HFNO, clinicians reported they consider coordination of respiration and swallowing.

Specifically they discussed potential interference with respiratory‐swallow synchronisation and timing of apnoea: “If someone's on high‐flow I just think okay they're on some increased respiratory support for starters, so all of the things you think about with that interfering with respiratory swallow synchronisation” (FG2); and “I'm wondering if they coordinate correctly… if they do the apnoea at the right time” (FG3).

#### 3.4.3 Safety and Efficiency

Changes in laryngo‐pharyngeal sensation were discussed as potentially occurring in the presence of HFNO: “*Their cough sensation might not be particularly reliable” (FG1)*. Safety concerns were also raised about pharyngeal and laryngeal residues being blown down into the airway by the flow: *“The other thing for me is if they are on high flow, high high flow, if there's a suspicion of pharyngeal residue whether or not that is at risk of being blown down into the airway” (FG2)*.

#### 3.4.4 Pressure Changes

Multiple participants discussed pressures during oropharyngeal swallowing as a consideration when patients are receiving HFNO. However, the impact of pressure was generalised and there was no consensus amongst participants for where ‘pressure’ impacts swallowing function. They referred to pressure changes in the oral cavity, pharynx, and larynx as illustrated in the following quotes: *“Swallow pressure and palatal closure” (FG2), “Laryngeal airway pressures with the blow” (FG3)* and *“That pressure from the high flow can even impact the closure and everything from an oral phase” (FG3)*.

### 3.5 **Category 5**:Current Knowledge and Practice Landscape

The current knowledge base and practice landscape in relation to assessing and managing adults on HFNO in a critical care environment was a significant topic discussed by participants in all three focus groups. Specifically, participants reported increasing referrals for swallowing assessments with patients receiving HFNO, limited staffing of SLT in critical care, and that a lack of evidence and research in this area is challenging.

#### 3.5.1 Frequency of HFNO use

Participants reported that in the past they were not assessing patients until they were off the high flow mask, weaned to low flow, or not receiving any oxygen support. They highlighted an increase in the use of HFNO in the last few years, and noted the opportunities for SLTs to assess swallowing earlier. The following quote is representative of this perspective: *“I think we're probably seeing swallowing earlier. People are able to eat and drink sooner with high‐flow nasal prongs being used way more often” (FG1)*.

#### 3.5.2 Staffing Challenges

Many participants talked about the challenges of staffing when working in critical care with adults on HFNO. The following quote captures this theme: *“SLT staffing challenges can be tricky because if you are down resources in your team, you're trying to keep these very complex and fragile patients safe” (FG2)*.

#### 3.5.3 Lack of Evidence and Research

All focus groups discussed the lack of evidence and research surrounding HFNO and oropharyngeal swallowing. They highlighted this challenge when working with patients on HFNO. The following quote is representative of participants’ concerns regarding the absence of robust evidence:

“One of the challenges is the lack of research that we have about the impact on swallowing, and you know that's why we're here isn't it […] we don't have conclusive evidence either way. We've got some studies that say that healthy normal swallows all are slightly altered, some studies that say it makes no difference so I think that would be helpful. That's a bigger challenge […] because we need the evidence” (FG2).

Participants also acknowledged that undertaking research in this population is difficult. The following quote illustrated this perceived barrier to conducting research with this cohort:

“I think it's an area that is so challenging in a sense that because of the aetiology of why they're needing the high‐flow and all of the other factors that are involved in it, it is a really hard one I guess to get […] robust evidence on” (FG3).

### 3.6 **Category 6**:Collaboration and Communication

Collaboration and communication were identified as intrinsic factors when working within an MDT. Participants also highlighted the necessity to seek patient perspectives of eating and drinking on HFNO.

#### 3.6.1 Working With the Multidisciplinary Team

Participants highlighted both challenges and opportunities when collaborating with the MDT to support patients on HFNO. A perceived challenge was that members of the MDT may not have considered the impact of HFNO on oropharyngeal swallowing. This is demonstrated clearly by the following quotes:

“I think when you explain to them [MDT] about the things that we might be concerned about they will seem a little bit surprised, so I think they just sort of assume that high‐flow is fairly innocuous” (FG2).

“I think they [MDT] probably don't know what risks come with the high‐flow they are probably more the other way ‘come and assess they look great […] they look like they'll be able to eat and drink’ and haven't considered what their high flow rate might do” (FG1).

One participant highlighted the complexity of risk management discussions with the MDT for this patient cohort: “The challenge is in that very careful nuanced dialogue about risk management that you need to have with the medical team and then the treating multidisciplinary team about risk versus benefit and quality of life versus protection versus enjoyment” (FG2).

Participants also discussed the opportunities that arise when working within the MDT and treating adults on HFNO, including having a collaborative approach and opportunities for learning. One participant stated, *“I think it's the opportunity for joint working as well—it's really lovely with this patient group there's lots of opportunities to learn […] I've learned a lot from the physiotherapist” (FG2)*.

#### 3.6.2 Patient Experience

In all three focus groups, the participants discussed they would like to know more about patients’ perspectives and experiences of eating and drinking while on HFNO. The following quote captures the essence of these discussions across the focus groups: *“It will be really interesting to get some perspectives, from a […] patient perspective around eating and drinking on high‐flow” (FG2)*.

## 4 Discussion

This is the first qualitative study exploring international SLTs’ experiences and opinions of assessing and managing patients on HFNO. It revealed that SLTs routinely assess and manage adults in critical care on HFNO. Potential impacts of HFNO on oropharyngeal swallowing were considered and participants raised concerns regarding the lack of evidence about the impact on swallowing. Most participants identified potential negative impacts to swallowing physiology including changes to laryngeal and pharyngeal sensation and pressures. Participants cautioned that higher flow rates may cause or lead to pharyngeal and laryngeal residues being blown into the airway. This may be associated with a higher risk for aspiration and pneumonia.

Participants reported that they consider oral, pharyngeal, and laryngeal pressure changes when assessing and managing adults on HFNO. Research demonstrates that use of HFNO creates positive nasopharyngeal airway pressure (Coghlan and Skoretz 2017). Researchers have postulated that the positive pressures in the oropharynx may negatively impact a person's ability to protect their airway during swallowing (Devlin and O'Bryan 2021). The Modified Barium Swallow Impairment Profile (MBSImp) is a standardised evaluation tool for VFSS which rates 17 components of swallowing (Martin‐Harris et al. 2008). Results from a study investigating the physiology of swallowing in a healthy adult population found that there was a statistically significant effect of flow rate on oral components of the MBSImp score, demonstrating that HFNO flow rate impacts the oral phase of swallowing (Eng et al. 2019).

Participants reported that they always consider patient status and trajectory when undertaking assessments. They highlighted that the critical care cohort are vulnerable, medically fragile, and typically have multiple comorbidities which may place them at a higher risk of aspiration and associated complications. This is supported by research that has identified numerous factors contributing to increased risk of swallowing difficulties in critical care populations. Factors include the presence of nasogastric or endotracheal tubes, multiple intubations, tracheostomy, reduced levels of consciousness due to medications, delirium, excessive sedation, gastroesophageal reflux, and ICU acquired weakness (Moore 2002, Macht et al. 2013).

In the uncharted territory of HFNO and dysphagia, and with concerns regarding the reliability of clinical bedside swallowing assessment, participants highlighted the importance of instrumental assessment due to the high risk of silent aspiration. This approach is supported by research that has shown that 50% of patients receiving HFNO with flow rates between 30–50 L/min were found to have silent laryngeal penetration or silent aspiration and cautioned SLTs about the potential effects of HFNO (Flores et al. 2019). Furthermore, a recent narrative review of HFNO and dysphagia also advise that clinicians take an individualised approach to managing dysphagia in patients receiving HFNO and recommend the use of instrumental assessment of swallowing with this population (Sutt and Wallace 2025).

While all participants reported that they have access to instrumental assessment, the use of instrumental assessment varied widely between countries. Participants from Australia and New Zealand reported that they frequently used instrumental assessments, stating it increased their confidence in making recommendations with this cohort. Similarly, a participant from the Republic of Ireland reported that their hospital's dedicated SLT critical care team has the capacity to administer FEES assessment within 24 h, resulting in timely assessment for their patients. However, not all hospitals and countries benefit from such ease of access to instrumental assessment; one participant from Italy reported frequent delays because passing an endoscope is usually performed by otolaryngologists. This variable access to instrumental assessment aligns with findings from an international survey which found that less than 40% of respondents from services in Ireland reported access to FEES assessment for critical care patients, and less than 60% of international respondents had access to FEES for their critical care units (Rowland et al. 2022). Fluctuations in medical status for this clinical population was also provided as a reason for not completing FEES by one participant from the United Kingdom. This is consistent with findings from the Dysphagia in Intensive Care Evaluation (DICE) survey which found that across 26 countries, few responding critical care units are using VFSS and FEES to diagnose dysphagia (Spronk et al. 2022).

A survey of feeding practices during HFNO therapy found that there was a mismatch between the opinions of SLTs and the rest of the MDT regarding commencement of oral intake during HFNO therapy (Charlton et al. 2023). They identified that 58.8% of physicians/advanced practice providers, respiratory therapists, and registered dietitians felt that patients do not require swallowing assessment before patients eat and/or drink while receiving HFNO, whereas 75.5% of SLT respondents were in favour of a clinical swallowing examination. These findings correlate with statements from the focus groups that members of the MDT believe HFNO is ‘innocuous’ and they may not consider the potential effects of HFNO on oropharyngeal swallowing. This may reflect the lack of current evidence about the impact of HFNO on oropharyngeal swallowing and highlights the need to conduct further research to enable SLTs to advocate and educate the MDT. Increasing the use of instrumental assessment such as FEES or VFSS would be a useful way to provide members of the MDT with accurate information about the impact of HFNO on swallowing function.

No participants used a dysphagia screening tool with patients receiving HFNO. Additionally, participants and authors were unaware of the existence of a validated screening tool for patients receiving HFNO. Early identification and treatment of swallowing disorders can prevent aspiration pneumonia and improve patients’ health outcomes (Sherman et al. 2021). Therefore, a valid and reliable screening tool to identify dysphagia and aspiration risk, suitability to commence oral intake, and when to refer to speech language therapy for patients receiving HFNO would be advantageous (See et al. 2016). Validating a dysphagia screening tool in patients receiving HFNO using a gold standard instrumental assessment (i.e., VFSS or FEES) would improve the tools available to manage these patients and our understanding of the impact of HFNO on swallowing and the potential for silent aspiration.

### 4.1 Limitations

Given the qualitative nature of the study, the researchers’ personal beliefs, values, or expectations may have influenced data analysis. In addition, when collecting data in focus groups there is the potential for conformity bias (Wong 2008). The use of online focus groups may have also influenced data collection. Although a virtual platform enabled participation of SLTs across multiple countries, it introduced the potential for limitations including reduced interpersonal interaction between participants and an inability to read nonverbal cues and body language compared with in‐person focus groups or interviews. Consequently, participant engagement, individual contributions, and openness of discussions may have been affected (Seitz 2016). The PI reminded participants that differences in opinions were welcomed and that discussions were confidential. Despite attempts to recruit a gender diverse sample, all study participants were female. This was unsurprising given the international gender profile of SLTs, with females representing 94.2% of the membership of 31 international speech‐language pathology associations (Campos et al. 2018). Participants were recruited through special interest groups, social media, and personal contacts, which limited the possibility of recruiting critical care SLTs from outside of these networks. Furthermore, the focus group took place in English which may have impacted recruitment from non‐English speaking countries.

### 4.2 Clinical Implications

There is currently limited robust evidence to guide assessment and management of swallowing in adults receiving HFNO in critical care. Findings from this study demonstrate variability in clinical practice and highlight uncertainty regarding assessment and management in this patient population. Some participants emphasised the value of instrumental assessment in supporting clinical decision making. This study identified that there are currently no standardised protocols or guidelines for the screening and comprehensive assessment for adults on HFNO. Further research is needed to guide the development of such protocols and guidelines to enable best practice.

### 4.3 Future Research

Current research provides SLTs and the MDT little guidance on managing swallowing in patients receiving HFNO. Evaluation of the physiological impact of different HFNO flow rates on swallowing function in both healthy individuals and patient populations would improve the safety and effectiveness of assessment and management of these patients.

Key considerations for future research include:

- Flow rates: Investigating the impact of varying HFNO flow rates on swallowing physiology.
- Instrumental assessment: Utilising VFSS and FEES to assess swallowing function objectively.
- Establishing the consistent use of a core outcome set would help ensure research is comparable.
- Screening: Validating a dysphagia screening tool for clinical use with this patient population.

Participants discussed the uncertainty regarding the influence of HFNO on sensation, laryngeal and oropharyngeal pressures, and airway closure. These areas may be worthy of investigation particularly in vulnerable patient populations. Improved understanding of these effects may assist SLTs in making evidenced based clinical decisions and tailoring recommendations to individual patients. Participants in all three focus groups also highlighted the need for further qualitative investigation of the patient experience of eating and drinking while on HFNO. This research may contribute to the evidence base required to inform future protocols and clinical guidance for swallowing management in patients receiving HFNO.

## 5 Conclusion

This focus group study provided insight into specialist critical care SLTs’ experiences and perceptions of assessment and management of swallowing in critical care patients receiving HFNO. These findings suggest that future research is needed to explore the relationship between HFNO flow rates and oropharyngeal swallowing and to establish best‐practice approaches for screening, assessment, and management of swallowing in this patient cohort.

## Funding

The authors received no financial support for the research, authorship or publication of this article.

Claire Mills is funded by an NIHR Postdoctoral Fellowship (NIHR507322). The views expressed are those of the author(s) and not necessarily those of the NIHR or the Department of Health and Social Care.

## Ethics Statement

Ethical approval for this research project was obtained on 02/03/2023 from Trinity College Dublin [HT19]. All participants provided informed consent prior to data collection.

## Conflicts of Interest

The authors declare that they have no conflicts of interest.

## Data Availability

The data that support the findings of this study are not available due to ethical and privacy restrictions.
